# Author Correction: Cyclic fasting bolsters cholesterol biosynthesis inhibitors’ anticancer activity

**DOI:** 10.1038/s41467-023-43184-4

**Published:** 2023-11-22

**Authors:** Amr Khalifa, Ana Guijarro, Silvia Ravera, Nadia Bertola, Maria Pia Adorni, Bianca Papotti, Lizzia Raffaghello, Roberto Benelli, Pamela Becherini, Asmaa Namatalla, Daniela Verzola, Daniele Reverberi, Fiammetta Monacelli, Michele Cea, Livia Pisciotta, Franco Bernini, Irene Caffa, Alessio Nencioni

**Affiliations:** 1 Department of Internal Medicine and Medical Specialties, University of Genoa, Viale Benedetto XV 6, 16132 Genoa, Italy; 2 Ospedale Policlinico San Martino IRCCS, Largo Rosanna Benzi 10, 16132 Genoa, Italy; 3 Department of Experimental Medicine, University of Genoa, Via Leon Battista Alberti 2, 16132 Genoa, Italy; 4 Department of Medicine and Surgery, University of Parma, 43125 Parma, Italy; 5 Department of Food and Drug, University of Parma, 43124 Parma, Italy; 6 Center of Translational and Experimental Myology, IRCCS Istituto Giannina Gaslini, 16147 Genoa, Italy

**Keywords:** Cancer metabolism, Colon cancer, Growth factor signalling, High-throughput screening, Pancreatic cancer

Correction to: *Nature Communications* 10.1038/s41467-023-42652-1, published online 31 October 2023

In this article, the author name Amr Khalifa was incorrectly written as By Amr Khalifa. The original article has been corrected.

