# Supplementary material for: Cyclic fasting bolsters cholesterol biosynthesis inhibitors’ anticancer activity
Source: Nat Commun. 2023 Oct 31;14:6951. doi: 10.1038/s41467-023-42652-1 (PMC10618279; doi:10.1038/s41467-023-42652-1)
Supplement: Supplementary file 1 — Supplementary Information [file 41467_2023_42652_MOESM1_ESM.pdf]

Supplementary Information

Cyclic fasting bolsters cholesterol biosynthesis inhibitors' anticancer activity

By Amr Khalifa[1,2], Ana Guijarro[1,2], Silvia Ravera[3], Nadia Bertola[3], Maria Pia Adorni[4], Bianca Papotti[5], Lizzia Raffaghello[6], Roberto Benelli[2], Pamela Becherini[1], Asmaa Namatalla[1], Daniela Verzola[1], Daniele Reverberi[2], Fiammetta Monacelli[1,2], Michele Cea[1,2], Livia Pisciotta[1,2], Franco Bernini[5], Irene Caffa[1,2*] and Alessio Nencioni[1,2*]

From the [1]Department of Internal Medicine and Medical Specialties, University of Genoa, Viale Benedetto XV 6, 16132 Genoa, Italy;
the [2]Ospedale Policlinico San Martino IRCCS, Largo Rosanna Benzi 10, 16132 Genoa, Italy;
the [3]Department of Experimental Medicine, University of Genoa, Via Leon Battista Alberti 2, 16132 Genoa, Italy
the [4]Department of Medicine and Surgery, University of Parma, 43125 Parma, Italy
the [5]Department of Food and Drug, University of Parma, 43124 Parma, Italy
and the [6]Center of Translational and Experimental Myology, IRCCS Istituto Giannina Gaslini, 16147 Genoa, Italy.

*These authors jointly supervised this work: Alessio Nencioni and Irene Caffa.

To whom correspondence should be addressed:

Dr. Alessio Nencioni, Department of Internal Medicine, University of Genoa, Viale Benedetto XV 6, 16132 Genoa, Italy; Phone: +39 010 353 8990; Fax +39 010 353 7989; Email: alessio.nencioni@unige.it

Dr. Irene Caffa, Department of Internal Medicine, University of Genoa, Viale Benedetto XV 6, 16132 Genoa, Italy; Phone: +39 010 353 7968; Fax +39 010 353 7989; Email: irene.caffa@unige.it

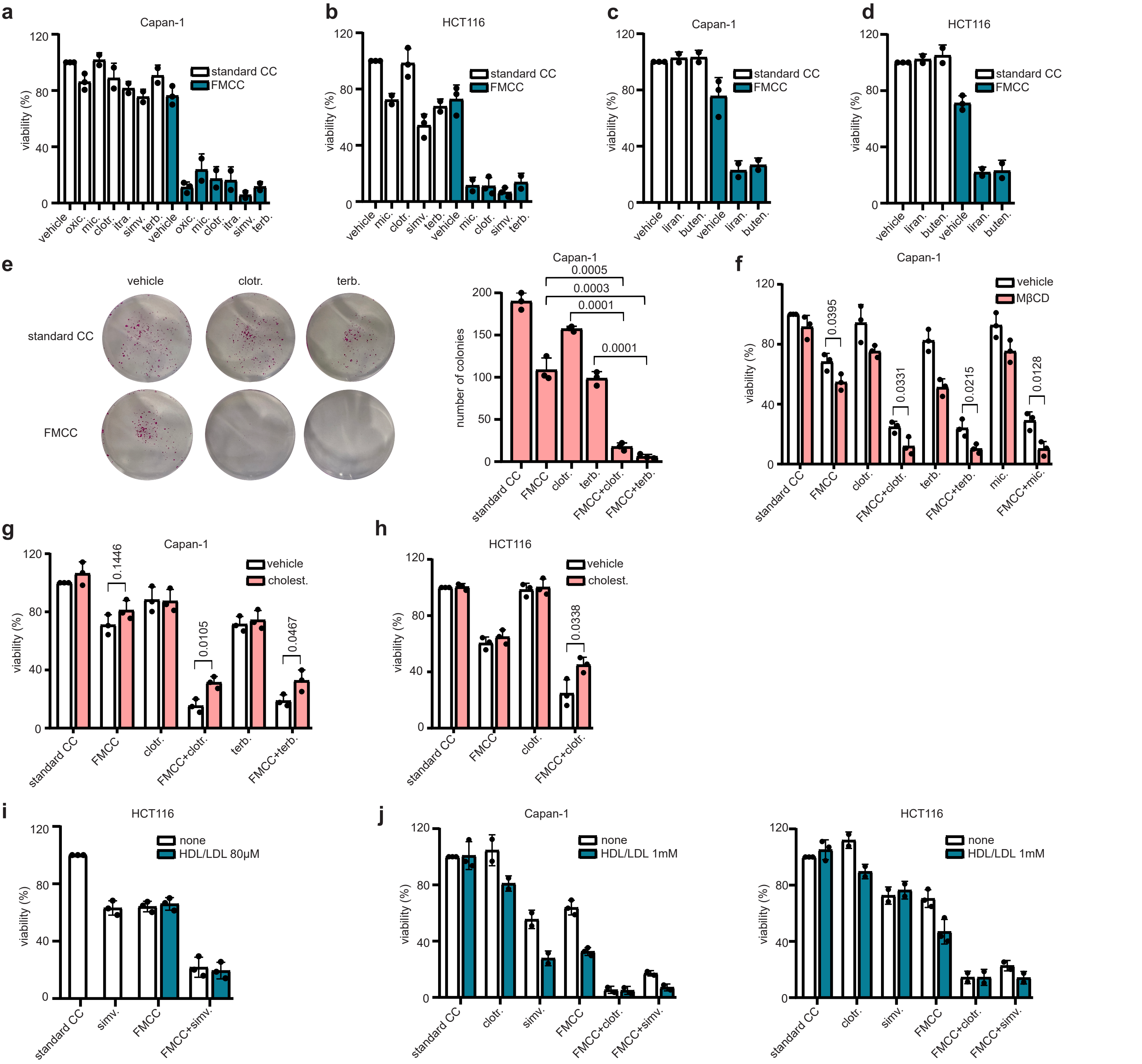

Supplementary Fig. 1. CBIs cooperate with FMCC to kill PDAC and CRC cells. **a,** Capan-1 cells were treated for 92h w/ or w/o FMCC. The indicated compounds (10 µM oxiconazole, miconazole or clotrimazole; 1 µM itraconazole; 30 µM simvastatin or terbinafine) or vehicle were added during the last 72h. Thereafter, cell viability was measured. **b**, HCT116 cells were treated for 92h w/ or w/o FMCC. The indicated compounds (10 µM miconazole or clotrimazole; 30 µM simvastatin or terbinafine) were added where indicated during the last 72h. Thereafter, cell viability was measured. **c**, **d**, Capan-1 cells and HCT116 were treated for 92h w/ or w/o FMCC. The indicated compounds (30 µM liranaftate or butenafine) or vehicle were added during the last 72h. Finally, cell viability was determined. **e**, Capan-1 cells were seeded in 6-well plates and cultured w/ or w/o FMCC for 24h. Then, vehicle, 15 µM clotrimazole or 30 µM terbinafine were added for 24h. Thereafter, cells were washed and cultured for additional 10 days in regular culture medium. Finally, the cell colonies were fixed and stained with sulforhodamine B, imaged and counted. **f**, Capan-1 cells were treated for 24h w/ or w/o FMCC. Then, 3.5 mM methyl-β-cyclodextrin (MβCD) w/ or w/o FMCC was added for 3h. Afterward, MβCD was replaced by vehicle or the indicated compounds (10 µM clotrimazole or miconazole, 25 µM terbinafine) in standard or FMCC for 72h before cell viability was determined. **g**, **h**, Capan-1 or HCT116 cells were treated for 96h w/ or w/o FMCC. During the last 72h the indicated compounds (15 µM clotrimazole or 25 µM terbinafine in Capan-1 cells; 10 µM clotrimazole in HCT116) were added in the presence or absence of 5 µg/ml cholesterol-MβCD. Afterwards, cell viability was quantified. i-j, Capan-1 and HCT116 cells were treated for 96h w/ or w/o FMCC. During the last 72h, 80 µM or 1 mM HDL/LDL cholesterol (at a 1:3 ratio) were added where indicated; 10 µM clotrimazole; 30 µM simvastatin. Thereafter, cell viability was measured. Data points are experimental replicates. Data are shown as mean±SD. Data were analysed by two-tailed Student's *t*-test. Source data are provided as a Source Data file.

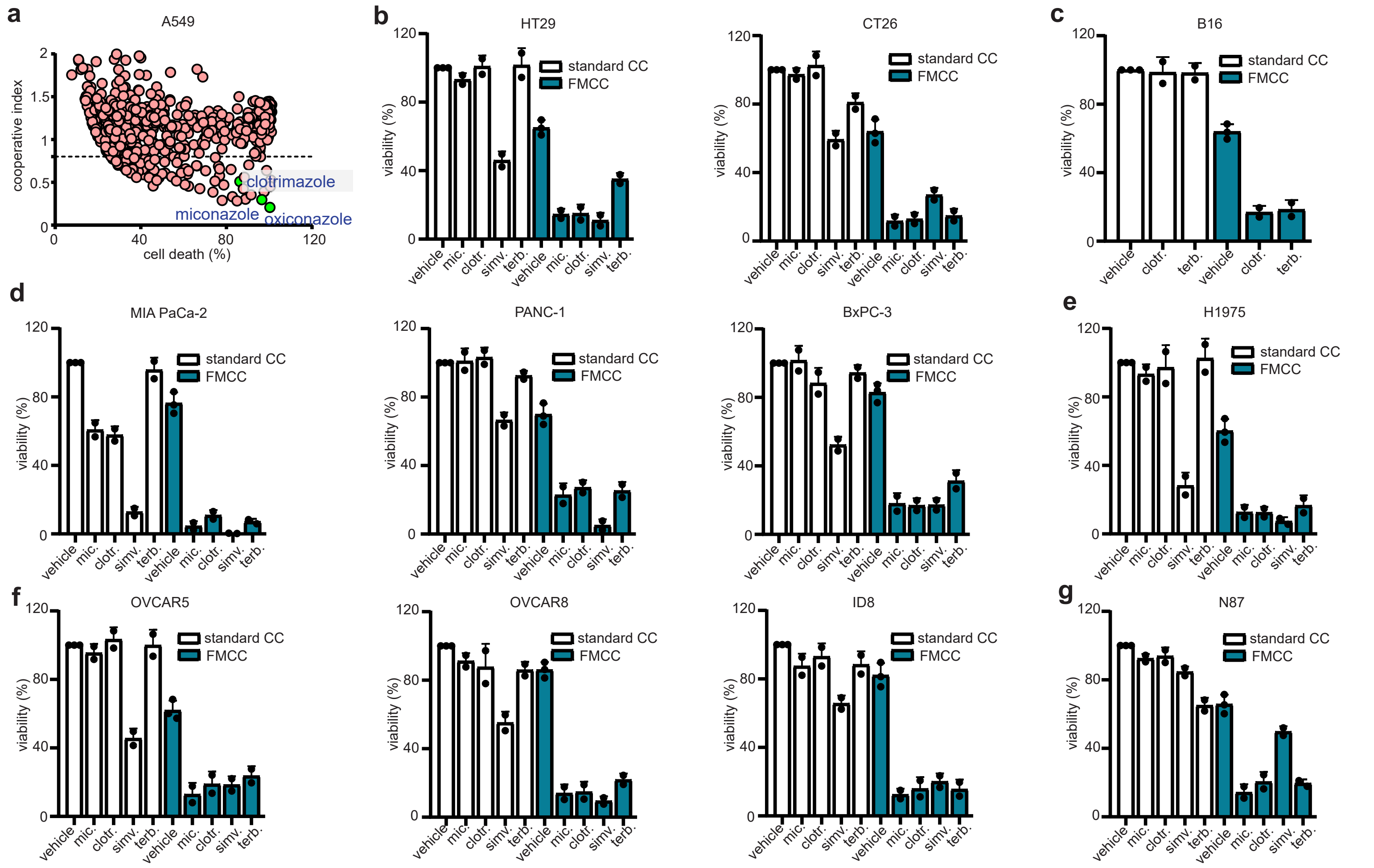

Supplementary Fig. 2. CBIs cooperate with FMCC to kill cancer cells of different histology. a, A549 non-small-cell lung cancer (adenocarcinoma) cells were plated in 96-well plates and allowed to adhere for 24h. Thereafter, cells were treated for 96h w/ or w/o FMCC and for the last 72h with libraries of bioactive or clinically approved compounds (Microsource Spectrum Collection and Selleck Preclinical/Clinical Compound Library) at a final 10 µM concentration. Finally, cell viability was determined. While a cooperative index <1 is typically considered to be indicative of a synergistic interaction between two agents, for higher stringency we chose a cooperative index ≤0.8 to define those drugs that synergistically interacted with FMCC. b-g, Colorectal (HT29 - human, CT26 - mouse), melanoma (B16 - mouse), pancreatic (MIAPaCa-2, PANC-1, BxPC3 - human), lung (H1975 - human), ovarian (OVCAR5, OVCAR8 - human; ID8 - mouse) and stomach (N87 - human) cancer cells were plated in 96-well plates and treated for 24h w/ or w/o FMCC. Thereafter, cells were stimulated with vehicle, 15 µM clotrimazole or miconazole or 30 µM terbinafine or simvastatin. Cell viability was determined 72h later. The drug screen (a) was performed once. Hit compounds (90) were retested twice and 18 of them were confirmed to synergize with FMCC in A549 cells. In b-g, data points are experimental replicates. In the histograms, data are shown as mean±SD. Source data are provided as a Source Data file.

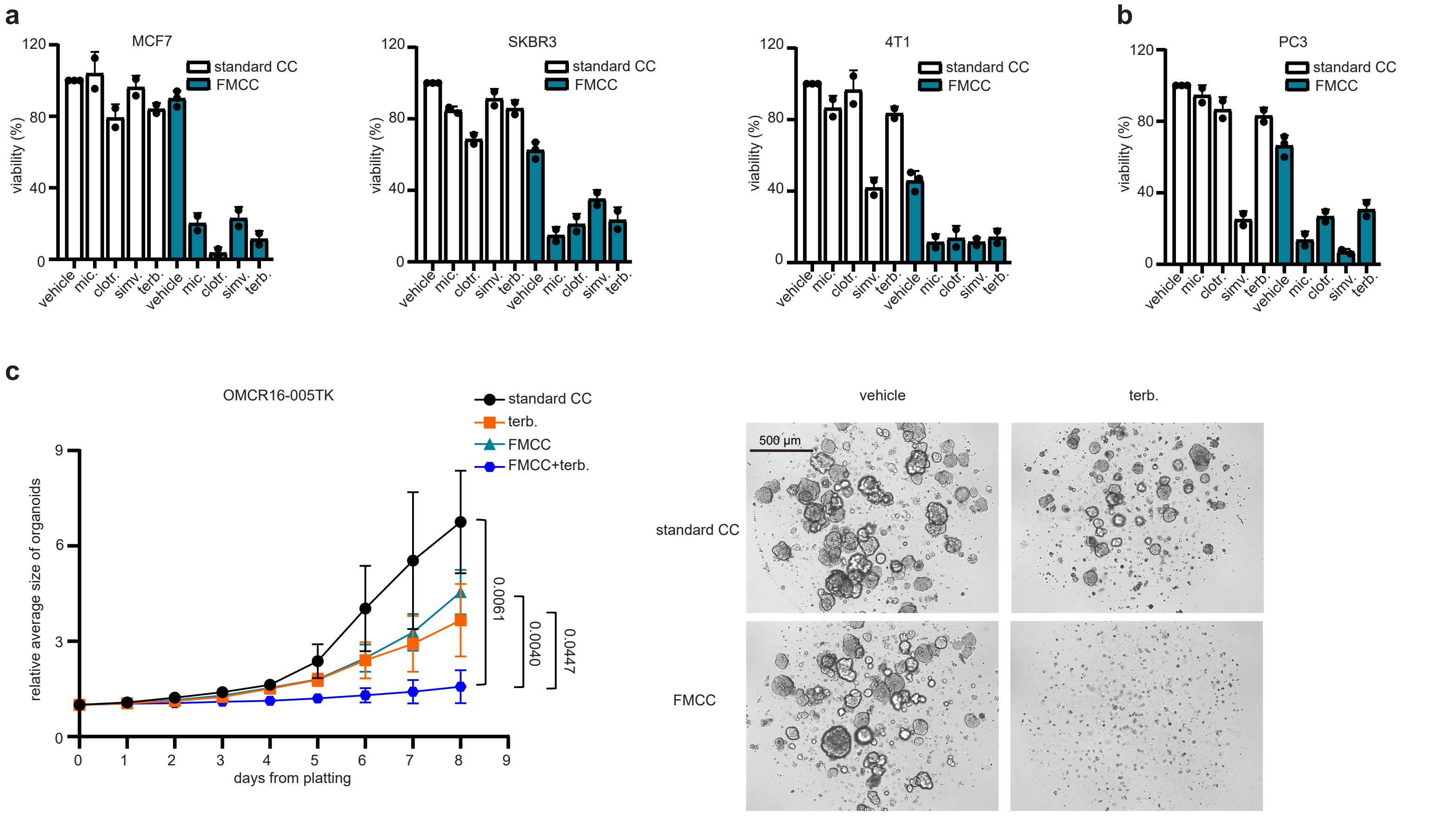

Supplementary Fig. 3. CBIs cooperate with FMCC to kill cancer cells of different histology and CRC organoids. **a-b**, Breast (MCF7, SKBR3 - human; 4T1 - mouse) or prostate (PC3 - human) cancer cells were plated in 96-well plates and treated for 24h w/ or w/o FMCC. Thereafter, vehicle, 15 µM clotrimazole or miconazole or 30 µM terbinafine or simvastatin were added during the last 72 h of incubation. Finally, cell viability was determined. **c**, The patient-derived CRC organoids, OMCR16-005TK, were treated for 192h w/ or w/o 20 µM terbinafine, FMCC or their combination. Organoids were imaged daily to estimate their size. The mean organoid area was calculated from the images from ten biological replicates using Image-J. The photographs in c show images of the organoids that were obtained on the last day of the experiments. A size bar is shown in the upper left image. In a, b, data points are experimental replicates; data are presented as mean±SD. In c, data are mean±SD of three experimental replicates; tumour organoid size from day 8 was analysed by two-tailed Student's *t*-test. Source data are provided as a Source Data file.

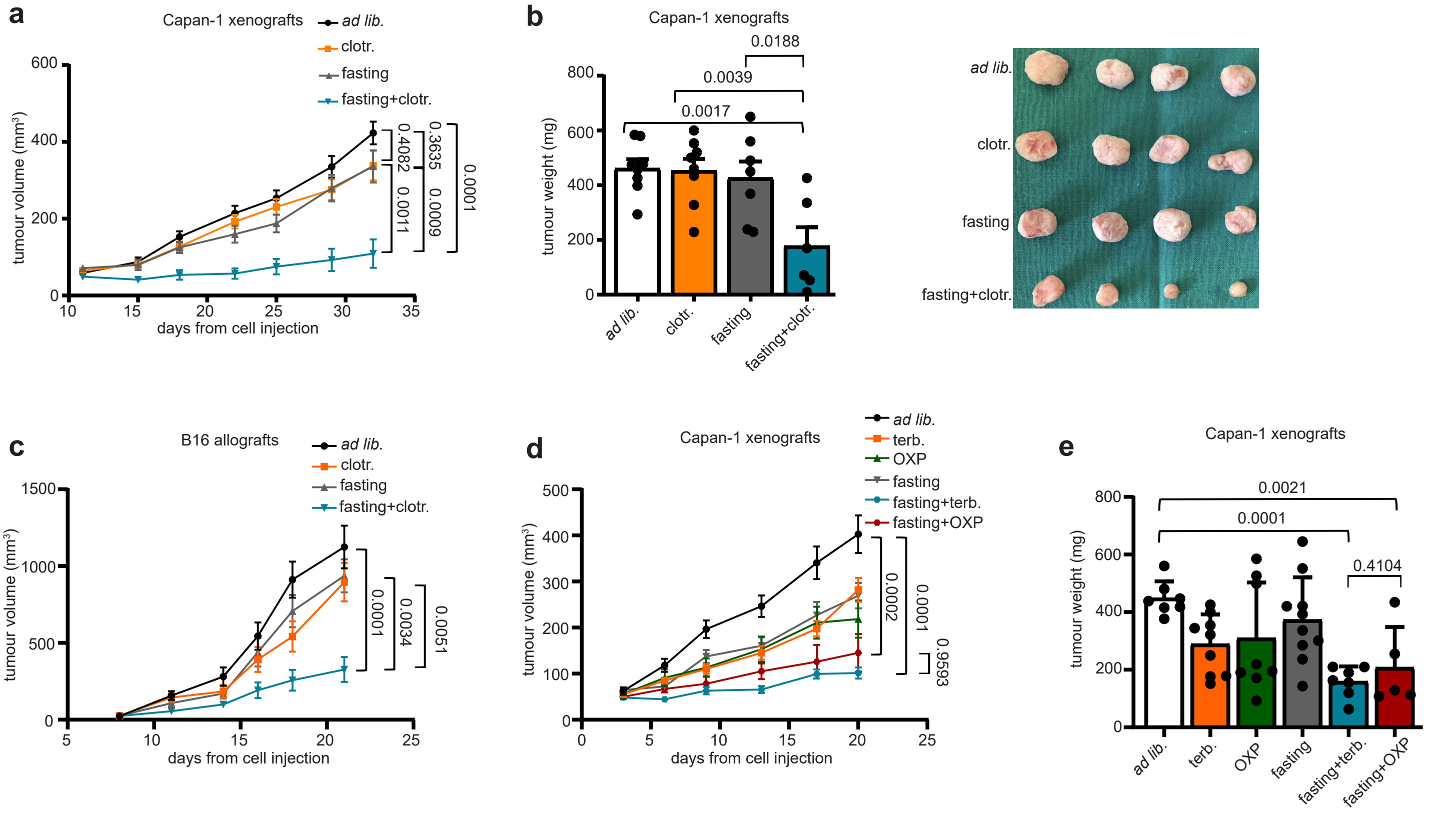

Supplementary Fig. 4. *In vivo* activity of cyclic fasting and CBIs against PDAC xenografts and mouse melanoma allografts. a, **b**, Capan-1 xenografts were established in 6-8-week-old female athymic nude mice. Once tumours were palpable, mice were randomized to be treated with *ad lib.* diet (*n*=8), weekly 48h water-only fasting (“fasting”; *n*=9), clotrimazole (60 mg/kg twice a week; *n*=8), combined fasting and clotrimazole (*n*=8). At the end of the experiment, tumours were weighted and imaged (b). **c**, Subcutaneous B16 mouse melanoma allografts were established in 6-8-week-old female C57BL/6 mice. Once tumours were palpable, mice were randomized to be treated with ad lib. diet (*n*=11), weekly 48h water-only fasting (“fasting”; *n*=14), clotrimazole (60 mg/kg twice a week; *n*=10), combined fasting and clotrimazole (*n*=14). Tumour volume was measured at the indicated time points. **d**, **e**, Capan-1 xenograft-bearing, 6-8-week-old female athymic nude mice were randomized to be treated with *ad lib.* diet (*n*=8), weekly 48h water-only fasting (“fasting”; *n*=11), terbinafine (40 mg/kg/day for five days a week; *n*=11), combined fasting and terbinafine (*n*=12), oxaliplatin (10 mg/kg, once a week; *n*=8), combined fasting plus oxaliplatin (*n*=11). Tumour volume was measured at the indicated time points and at the end of the experiment (**d**) and the excised tumours were weighted (**e**). In d and e, the data from mice that were treated with *ad lib.* diet, fasting, terbinafine or fasting plus terbinafine were already presented in Fig. 3e-f. They are shown here again to allow comparison with the treatment group and fasting+oxaliplatin (these treatment groups were all part of the same experiment). In a, c and d, *n* indicates the number of tumours per treatment group. In b and e, data points are biological replicates (they represent single tumours). Data are shown as mean±SEM in a-d and as mean±SD in e. In a, c and d data were analysed by one-way ANOVA with Tukey post-test. In b and e, data were analysed by two-tailed Student’s *t*-test. Source data are provided as a Source Data file.

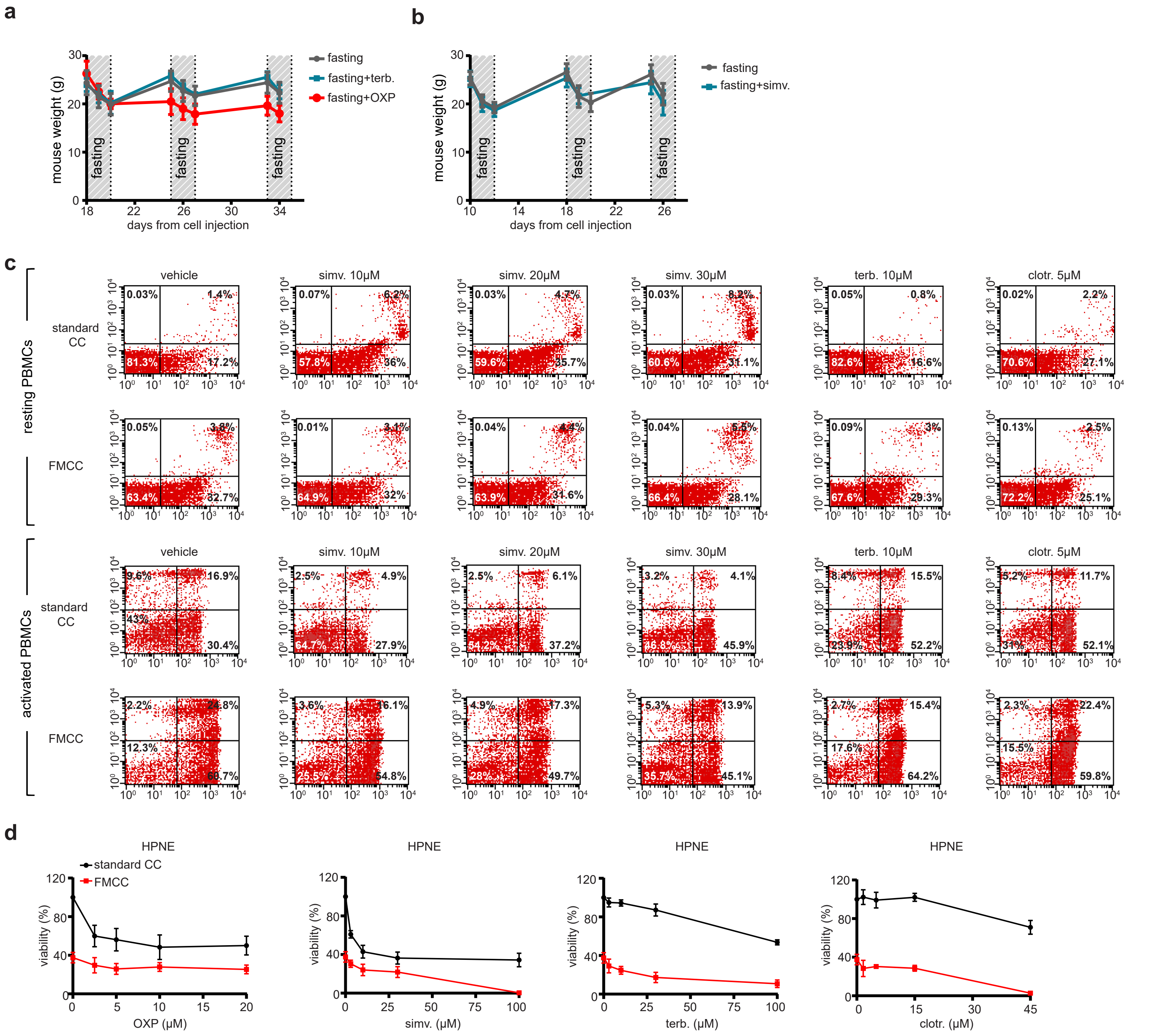

Supplementary Fig. 5. Toxicity of CBIs, fasting and their combination. a, **b**, Mouse weight of animals from Supplementary Fig. 4d (mice that were treated with fasting, fasting+terbinafine or fasting+oxaliplatin) and from Fig. 2f (mice that were treated with fasting or fasting+simvastatin) was monitored throughout treatment. **c**, PBMC from a buffy coat obtained from a healthy donor were plated in 48-well plates and were cultured for 72h in the presence or absence of FMCC w/ ("activated PBMCs") or w/o ("resting PBMCs") 2 µg/ml PHA, simvastatin, terbinafine or clotrimazole. Thereafter, apoptosis was quantified by staining with Annexin-V-FITC and propidium iodide and by subsequent flow cytometry. A figure showing the gating strategy that was adopted in these FACS analyses is shown at the end of this file. **d**, HPNE cells were treated for 96h w/ or w/o FMCC and for the last 72h w/ or w/o oxaliplatin, terbinafine or clotrimazole at the indicated concentrations. Finally, cell viability was determined. In a and b data are shown as mean±SD. In a, the mice treated with fasting, fasting+terbinafine or fasting+oxaliplatin were six in each group.In b, the mice treated with fasting or fasting+simvastatin were five and six, respectively. In c, one representative experiment out of three is presented. In d, data are means±SD of three experimental replicates. Source data are provided as a Source Data file (a, b, d).

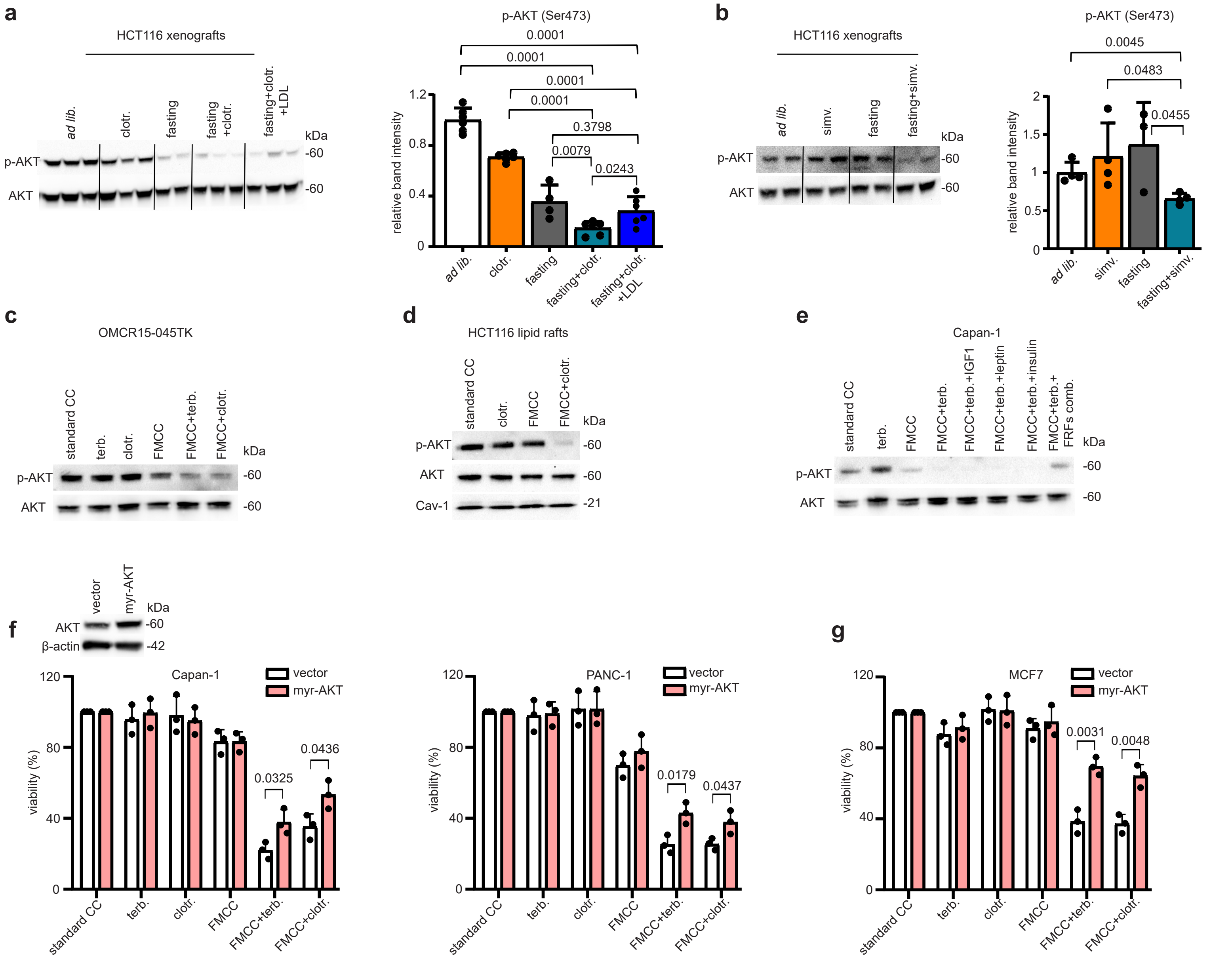

Supplementary Fig. 6. Combined fasting and CBIs slow tumour growth by inhibiting AKT. a, **b**, Phosphorylated (Ser473) AKT and total AKT were detected by Western blotting in HCT116 xenografts from the experiments shown in Fig. 2h-j (a; mice were treated with either *ad lib.* diet, clotrimazole, weekly fasting, clotrimazole+fasting or clotrimazole+fasting+LDLs) and in Fig. 2f, g (b; mice were treated with either *ad lib.* diet, simvastatin, weekly fasting or simvastatin+fasting). Phosphorylated (Ser473) AKT band intensity was normalized to that of total AKT. **c**, OMCR15-045TK CRC organoids were treated for 24h with 15 µM clotrimazole or with 20 µM terbinafine w/ or w/o FMCC. Then, cells were used for protein lysate generation and phosphorylated (Ser473) and total AKT were detected by immunoblotting. **d**, Western blot analysis of phosphorylated (Ser473) AKT, total AKT and Caveolin-1 from plasma membrane-derived lipid rafts isolated from HCT116 cells treated for 48h w/ or w/o FMCC. 15 µM clotrimazole was added during the last 24h where indicated. **e**, Capan-1 cells treated for 48h w/ or w/o FMCC, IGF1 (5 ng/ml), leptin (50 ng/ml), insulin (500 pM), or IGF1+leptin+insulin. 20 µM terbinafine was added during the last 24h where indicated. Then, cells were used for protein lysate generation and phosphorylated and total AKT were detected by immunoblotting. **f**, **g**, Capan-1 and PANC-1 PDAC cells (**f**), as well as MCF7 breast cancer cells (**g**) were transduced with myr-AKT or with a control vector. Thereafter, cells were treated for 96h w/ or w/o FMCC. During the last 72h, 25 µM (in PDAC cells) or 10 µM (in MCF7 cells) terbinafine; or 10 µM (in PDAC cells) or 5 µM (in MCF7 cells) clotrimazole were added. Finally, cell viability was quantified. In the Western blot band quantifications from a and b, data points are biological replicates (they represent single tumours); samples derive from the same experiment and blots were processed in parallel. In f and g, data points are experimental replicates. In c-e, one out of three independent experiments is presented. In the histograms from a, b, f, g, data are shown as mean±SD. *p* values were calculated by two-tailed Student's *t*-test. Source data are provided as a Source Data file and at the end of this file (uncropped Western blots).

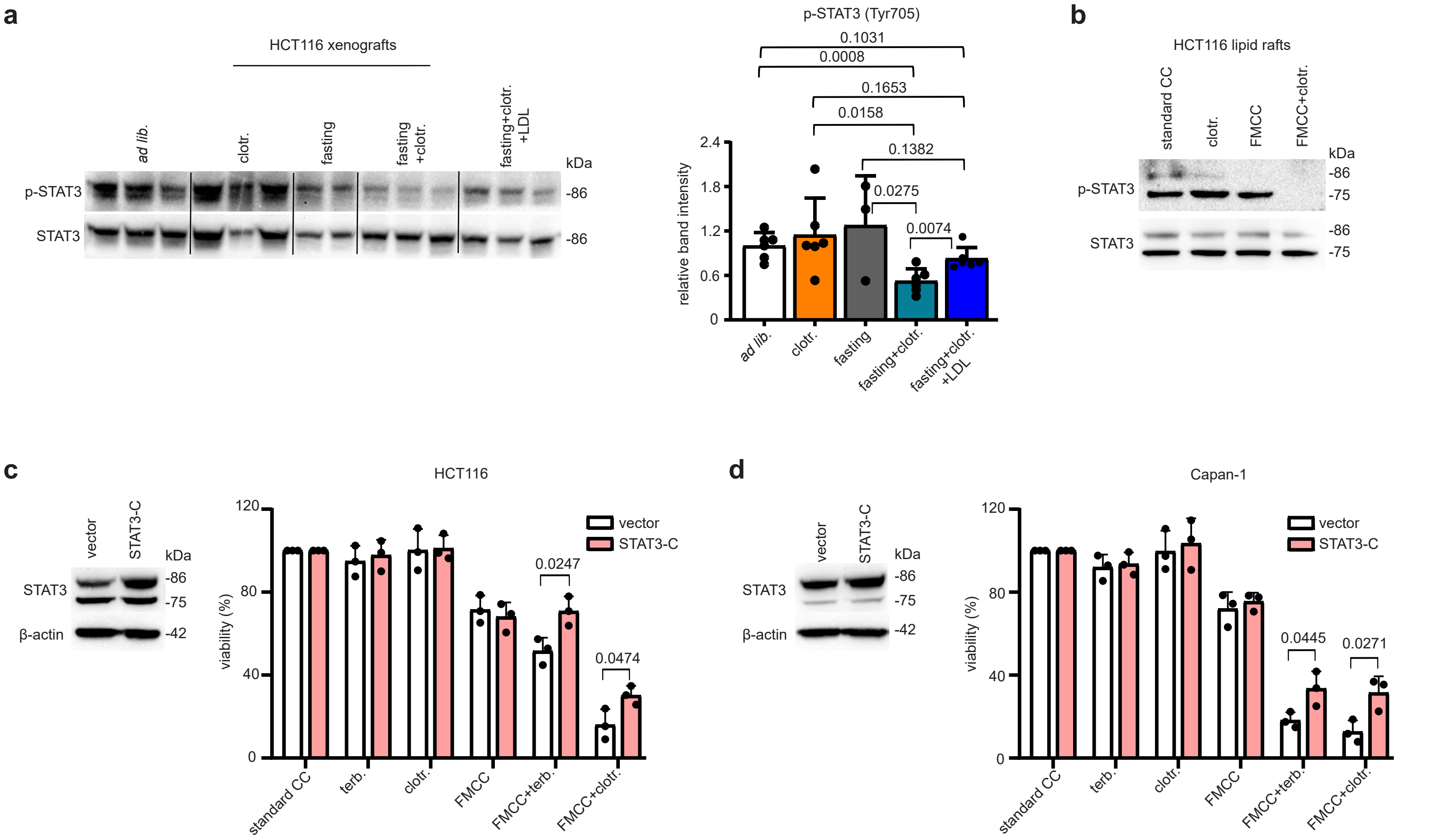

Supplementary Fig. 7. Fasting and CBIs cooperate to slow tumour growth through STAT3 inhibition. a, Phosphorylated (Tyr705) and total STAT3 were detected by Western blotting in HCT116 xenografts from the experiments shown in Fig. 2h-j (mice were treated with either ad lib. diet, clotrimazole, weekly fasting, clotrimazole+fasting or clotrimazole+fasting+LDLs). Phosphorylated STAT3 bands were quantified and normalized to the respective total STAT3 bands. **b**, HCT116 cells were treated for 24h w/ or w/o FMCC. 15 µM clotrimazole was added during the subsequent 24h where indicated. Thereafter, plasma membrane-derived lipid rafts were isolated and phosphorylated and total STAT3 were detected by Western blotting. **c**, HCT116 cells were transduced with constitutively active STAT3 (STAT3-C) or with a control vector. Cells were then plated in 96-well plates and treated for 24h w/ or w/o FMCC. Thereafter, vehicle, 15 µM clotrimazole or 10 µM terbinafine were added where indicated. 72h later, cell viability was quantified. **d**, Capan-1 cells that were transduced with a control vector or STAT3-C were plated and then treated for 24h w/ or w/o FMCC. Thereafter, cells were treated w/ or w/o 15 µM clotrimazole or 30 µM terbinafine. Cell viability was determined after 72h. In the Western blot band quantification from a, samples derive from the same experiment; blots were processed in parallel; data points are biological replicates (they represent single tumours). In the histograms from c, d, data points are experimental replicates. The Western blots from c and d are one representative experiment out of two. In the histograms from a, c and d, data are shown as mean±SD. *p* values were calculated by two-tailed Student's *t*-test. Source data are provided as a Source Data file (a, c, d - histograms data) and at the end of this file (uncropped Western blots).

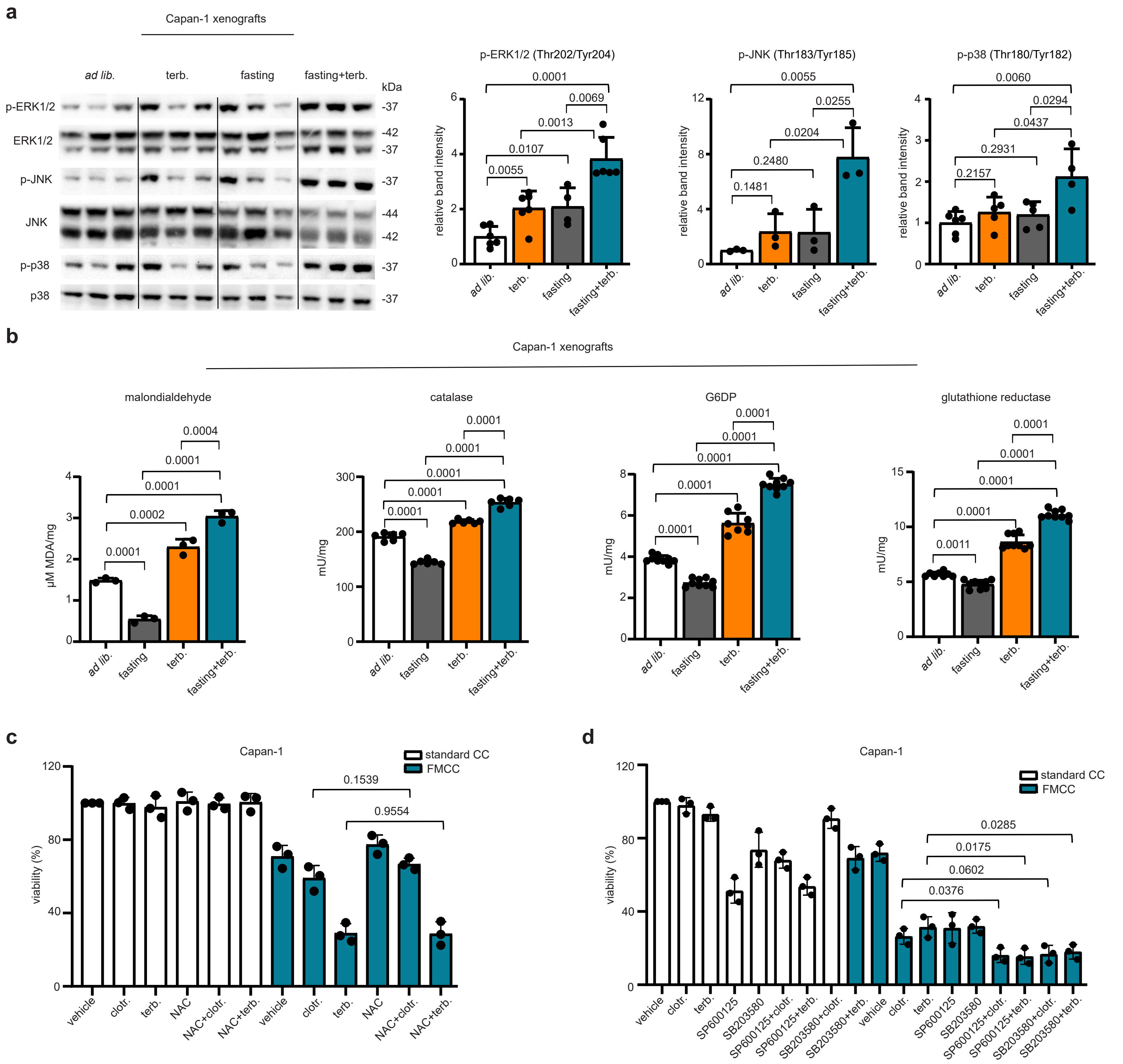

Supplementary Fig. 8. Fasting and CBIs increase ERK, p38 and JNK MAPK phosphorylation in Capan-1 xenografts. **a**, Phosphorylated (Thr202/Tyr204) and total Erk1/2, phosphorylated (Thr183/Tyr185) and total SAPK/JNK and phosphorylated (Thr180/Tyr182) and total p38 MAPK were detected by immunoblotting in Capan-1 xenografts from the experiments presented in Fig. 2a (mice were treated with either *ad lib.* diet, terbinafine, weekly fasting, terbinafine+fasting). Band intensity of the phosphorylated proteins was normalized to the band intensity of the corresponding total protein. **b**, Malondialdehyde (MDA) levels and the enzymatic activity of catalase, glucose-6-phosphate dehydrogenase (G6PD) and glutathione reductase were measured in Capan-1 xenografts from the experiments shown in Fig. 2a. **c**, **d**, Capan-1 cells were treated for 24h w/ or w/o FMCC. Thereafter, 15 μM clotrimazole or 30 μM terbinafine were added in the presence or absence of 5 mM N-acetylcysteine (NAC) (**c**), the JNK inhibitor, SP600125 (10 μM), or of the p38 MAPK inhibitor, SB203580 (20 μM) (**d**), w/ or w/o FMCC. Cell viability was measured 72h later. In the Western blot band quantification from a, samples derive from the same experiment; blots were processed in parallel; data points are biological replicates (they represent single tumours). In b, data points are biological replicates (single tumours). In c and d, data points are experimental replicates. Histogram data (a-d) are shown as mean±SD. *p* values were calculated by two-tailed Student's *t*-test. Source data are provided as a Source Data file (a, b, c, d - histograms data) and at the end of this file (uncropped Western blots).

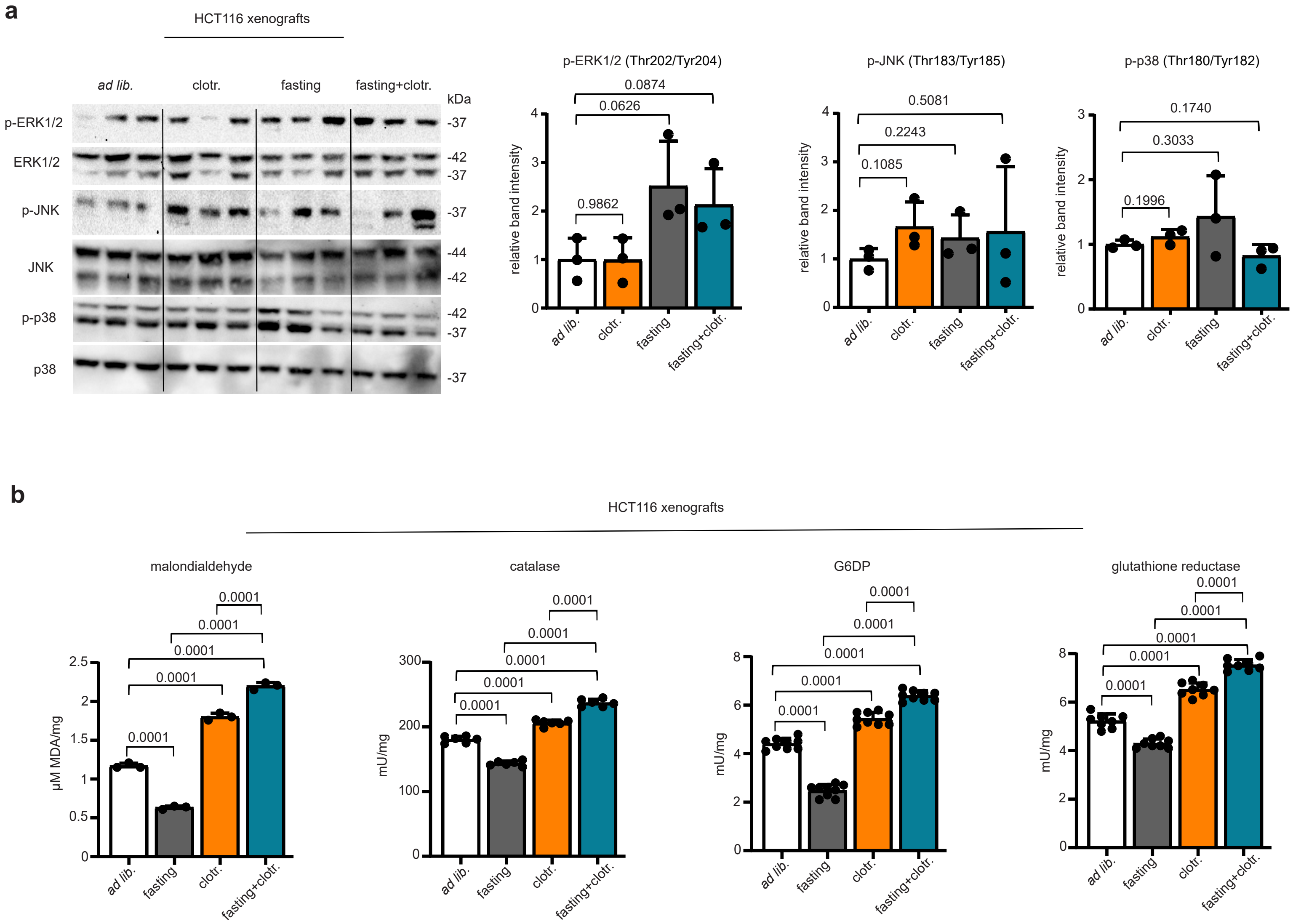

Supplementary Fig. 9. Effects of fasting and CBIs on ERK, p38 and JNK MAPK phosphorylation and on ROS signaling in HCT116 xenografts. a, Phosphorylated (Thr202/Tyr204) and total Erk1/2, phosphorylated (Thr183/Tyr185) and total SAPK/JNK and phosphorylated (Thr180/Tyr182) and total p38 MAPK were detected by immunoblotting in HCT116 xenografts from the experiments presented in Fig. 2h (mice were treated with either *ad lib.* diet, clotrimazole, weekly fasting, clotrimazole+fasting). Band intensity of the phosphorylated proteins was normalized to the band intensity of the corresponding total protein. **b**, Malondialdehyde (MDA), catalase, glucose-6-phosphate dehydrogenase (G6PD) and glutathione reductase levels were measured in HCT116 xenografts from the experiments shown in Fig. 2h. In a (histograms) and b, data points are biological replicates (they represent single tumours). In the histograms, results are presented as mean±SD and p values were calculated by two-tailed Student's t-test. Source data are provided as a Source Data file (a, b - histograms) and at the end of this file (uncropped Western blots).

Supplementary Table 1. Synergistic enhancement of drug anti-tumour activity through FMCC in PK9 PDAC cells.

Drug name	Cell death w/FMCC (%)	Cooperative index
Clotrimazole	83.2	0.80
Danazol	96.1	0.80
Guanabenz acetate	96.4	0.80
CCT129202	89.3	0.80
Imipramine hydrochloride	94.1	0.78
Chlorhexidine	94.5	0.79
Pizotyline malate	48.9	0.79
U0126-etoh	67.1	0.78
Hexylresorcinol	95.0	0.78
Dopamine hydrochloride	95.1	0.78
Dienestrol	92.8	0.78
Isoproterenol hydrochloride	97.2	0.76
Diflunisal	89.0	0.76
Benazepril hydrochloride	47.0	0.75
Desipramine hydrochloride	91.1	0.74
Chlorpromazine	92.2	0.73
Dicyclomine hydrochloride	95.2	0.73
Chlorcyclizine hydrochloride	93.0	0.72
Clomiphene citrate	94.5	0.71
Tamoxifen citrate	100	0.61
Candesartan cilextil	62.9	0.60
Vatalanib	72.9	0.53
Toremifene Citrate	101.1	0.51
Amlodipine besylate	90.5	0.46
Miconazole nitrate	86.8	0.41
Oxiconazole nitrate	100.2	0.35

Supplementary Table 1 shows the drugs which were found to synergistically interact with FMCC to kill PK9 PDAC cells in a drug screening. PK9 pancreatic cancer cells were plated in 96-well plates and allowed to adhere for 24h. Thereafter, cells were treated for 96h w/ or w/o FMCC and for the last 72h with libraries of bioactive or clinically approved compounds at a final 10 µM concentration before cell viability was detected. A cooperative index <0.8 was arbitrarily chosen to select for drugs that synergistically interacted with FMCC.

Supplementary Table 2. Complete blood counts and serum biochemistry in mice treated with clotrimazole, weekly 48h fasting or their combination w/ or w/o add back of LDL-cholesterol.

blood test	***ad lib.* diet**	**clotr.**	**fasting**	**fasting+ clotr.**	**fasting+ clotr.+ LDL**
	**n=4**	**n=4**	**n=3**	**n=4**	**n=3**
hemoglobin (g/dL)	14.25±0.92	14.7±1<br>n.s.	13.9±1.99<br>n.s.	12.07±3.69<br>n.s.	12.46±4.6<br>n.s.
hematocrit (%)	47.35±2.47	49.05±2.49	44.96±6.88	42±12.35	41.56±15.05
mean corpuscular volume (fL)	50.22±1.02	50.17±1.25<br>n.s.	50.23±1.77<br>n.s.	54.75±1.61<br>*p*=0.005	50.86±4.07<br>n.s.
white blood cells (K/µL)	8.59±3.76	8.69±2.82<br>n.s.	2.44±1.14<br>*p*=0.041	8.6±2.07<br>n.s.	5.15±3.54<br>n.s.
neutrophils (K/µL)	3.49±1.15	3.13±2.78<br>n.s.	1.42±0.48<br>*p*=0.03	5.26±0.95<br>n.s.	3.12±1.54<br>n.s.
lymphocytes (K/µL)	3.46±1.48	3.45±0.73<br>n.s.	0.86±0.58<br>*p*=0.032	2.61±1.09<br>n.s.	1.74±1.75<br>n.s.
platelets (K/µL)	1164±118	1709±432<br>n.s.	918±236<br>n.s.	1755±661<br>n.s.	1543±301<br>n.s.
red blood cells (M/µL)	9.43±0.49	9.78±0.5<br>n.s.	8.94±1.23<br>n.s.	7.71±2.39<br>n.s.	8.2±3.06<br>n.s.
creatine kinase (U/L)	350±123	442±183<br>n.s.	1717±1041<br>*p*=0.043	1143±724<br>n.s.	596±94<br>*p*=0.031
urea (mg/dL)	57.25±7.58	50.25±8.8<br>n.s.	51±21.63<br>n.s.	67.5±9.88<br>n.s.	62.66±25.69<br>n.s.
aspartate aminotransferase (U/L)	150.25±38.49	154±37.91<br>n.s.	391.66±180.37<br>*p*=0.044	269.25±191.21<br>n.s.	222.33±38.13<br>n.s.
alanine transaminase (U/L)	47.5±20.68	37.5±5.44<br>n.s.	79.66±44.29<br>n.s.	111.25±35.98<br>*p*=0.029	48.33±11.5<br>n.s.

Mice were treated with *ad lib*. diet, weekly 48h water-only fasting, clotrimazole (60 mg/kg twice a week), combined fasting plus clotrimazole, or fasting, clotrimazole and human LDL (0.25 mg/mouse twice a week coupled with fasting). Total blood and serum were collected at mouse sacrifice, i.e. after 20 days of treatment. The tumour data from this experiment are available in Fig. 2h-j. *p* values (compared to *ad lib.* diet) were calculated by two-tailed *t*-test. n.s.: non-sinificant. *n* indicates the number of mice per treatment group whose whole blood and serum were analysed for haematological and biochemical toxicity markers.

Supplementary Table 3. Serum biochemistry from mice treated with simvastatin, weekly 48h fasting, or their combination.

blood test	***ad lib.* diet**	**simvastatin**	**fasting**	**fasting+ simvastatin**
	**n=3**	**n=4**	**n=4**	**n=5**
creatinine (mg/ml)	0.46±0.05	0.43±0.05<br>n.s.	0.34±0.05<br>*p*=0.015	0.34±0.08<br>*p*=0.032
urea (mg/dL)	41±7.58	40.8±8.8<br>n.s.	43.8±21.63<br>n.s.	67.6±9.88<br>n.s.
aspartate aminotransferase (U/L)	160.3±25.8	155±19.6<br>n.s.	268.5±28.1<br>*p*=0.004	613.4±325.8<br>*p*=0.035
alanine transaminase (U/L)	40.3±25.7	27.5±2.9<br>n.s.	43±11.9<br>n.s.	104.9±19.0<br>*p*=0.027
creatine kinase (U/L)	571.3±217.2	624.8±321<br>n.s.	1,335±325,7<br>*p*=0.014	5,690.6±4,942.7<br>n.s.

Mice were treated with *ad lib*. diet, weekly 48h water-only fasting, simvastatin (80 mg/kg/day), combined fasting plus simvastatin. Total blood and serum were collected at mouse sacrifice, i.e. after 17 days of treatment. The tumour data from this experiment are available in Fig. 2f, g. *p* values (compared to *ad lib.* diet) were calculated by two-tailed *t*-test. n.s.: non-significant. *n* indicates the number of mice per treatment group whose whole blood and serum were analysed for blood counts and for standard biochemistry markers.

Supplementary Table 4. QPCR primer sequences

GENE	FORWARD PRIMER	REVERSE PRIMER	PROVIDER
**ABCG1**	ATCTCCTATGTCAGGTATGG	AGGGAGATGAAGAAAATCCC	MERK/Sigma-Aldrich
**ACAT1**	TCCAATTGGGATGTCTGG	CTCCTCCATTGCAAATACTG	MERK/Sigma-Aldrich
**IDI1**	AATAAACACTAACCACCTCG	CTCGATGCAATAATCCTTTCTC	MERK/Sigma-Aldrich
**FDPS**	GTCAGATTCACTGAAAAGAGG	GTCAAGGTAATCATCCTGAATC	MERK/Sigma-Aldrich
**LSS**	GCTGAACATGAGAAATCCAG	TAGTCAATCATGATGTCCCC	MERK/Sigma-Aldrich
**HMGCS1**	TTGGCTTCATGATCTTTCAC	AATTTAACATCCCCAAAGGC	MERK/Sigma-Aldrich
**DHCR24**	ACACCAAGAAACAGATTGTC	TACTTGTGGGATGATGACTC	MERK/Sigma-Aldrich
**DHCR7**	CAAAGCCAAGAGTCTAGATG	AAGTAGTAGACGATGAAGGG	MERK/Sigma-Aldrich
**LDLR**	TACAGCTACCCCTCGAGAC	CAGGCAATGCTTTGGTCTTCT	Invitrogen
**ACTB**	CCTCGCCTTTGCCGATCC	CGCGGCGATATCATCATCC	Invitrogen
**HPRT1**	TGGCGTCGTGATTAGTGATG	GCAAGACGTTCAGTCCTGTC	Invitrogen

Gating Information for Supplementary Figure 5c

a, b, Gating on the lymphocyte population that was utilized for the FACS analysis of Annexin-V and propidium iodide fluorescence in «resting» (a) and in activated (b) PBMCs, respectively.

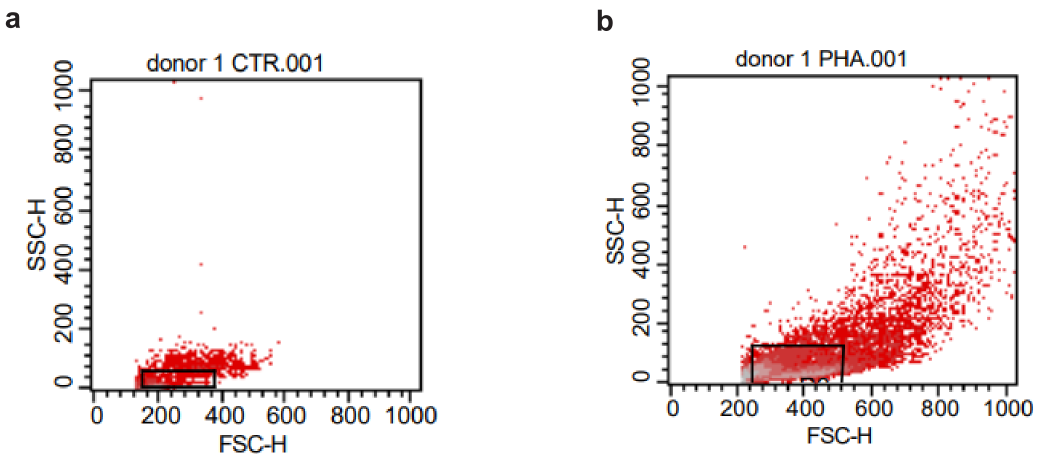

Uncropped Western blots from Supplementary Figure 6

Supplementary Fig. 6a

HCT116 xenografts

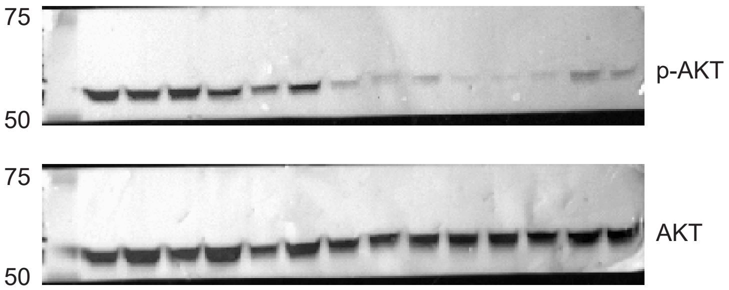

p-AKT and AKT were detected on the same membrane

Supplementary Fig. 6b

HCT116 xenografts

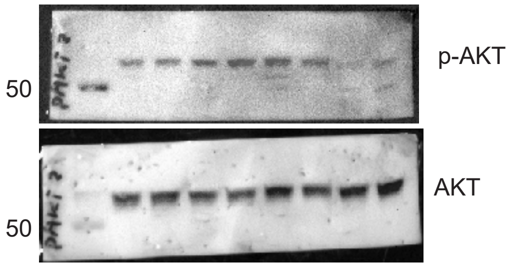

p-AKT and AKT were detected on the same membrane

Supplementary Fig. 6c

OMCR15-045TK

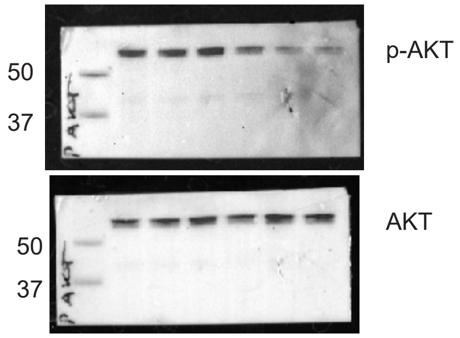

p-AKT and AKT were detected on the same membrane

Supplementary Fig. 6d

HCT116 lipid rafts

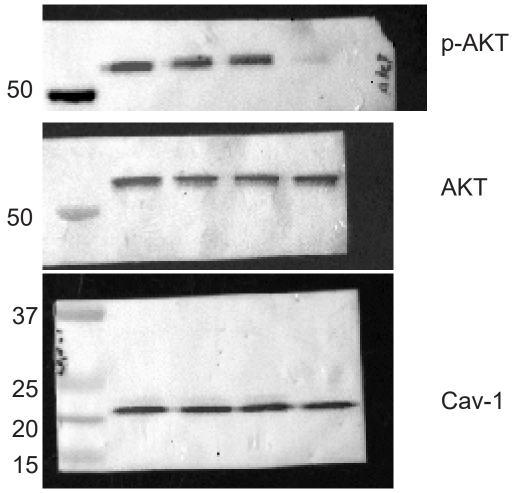

p-AKT and Cav-1 were detected on the same membrane, while AKT was detected on different membrane

Supplementary Fig. 6e

Capan-1 xenografts

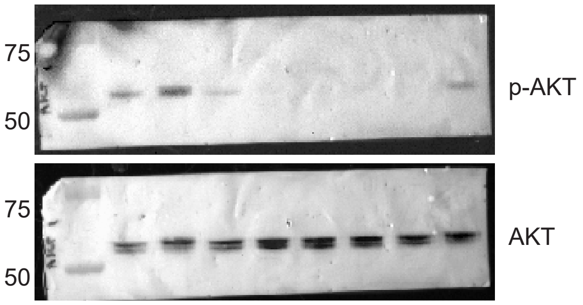

p-AKT and AKT were detected on the same membrane

Uncropped Western blots from Supplementary Figure 7-9

Supplementary Fig. 7a

HCT116 xenografts

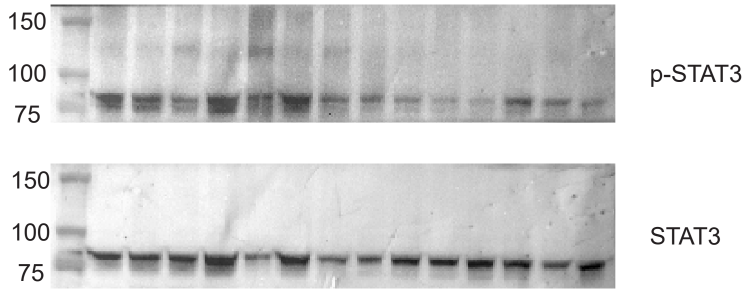

p-STAT3 and STAT3 were detected on the same membrane

Supplementary Fig. 7b

HCT116 lipid rafts

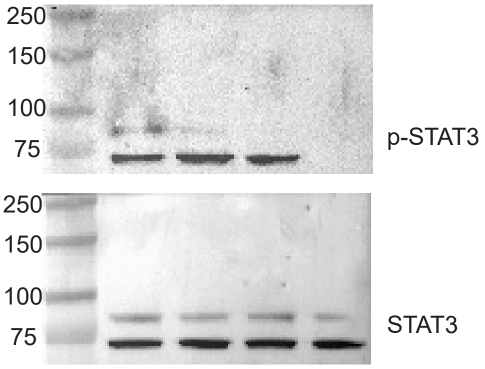

p-STAT3 and STAT3 were detected on different membranes

Supplementary Fig. 8a

Capan-1 xenografts

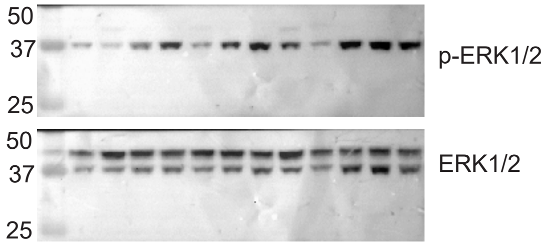

p-ERK1/2 and ERK1/2 were detected on the same membrane

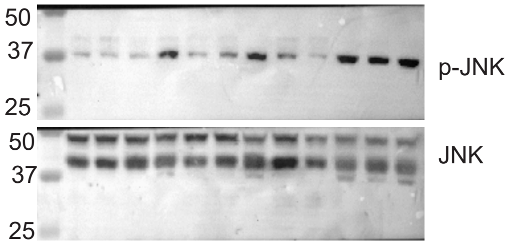

p-JNK and JNK were detected on the same membrane

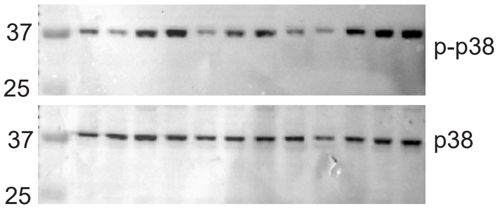

p-p38 and p38 were detected on the same membrane

Supplementary Fig. 9a

HCT116 xenografts

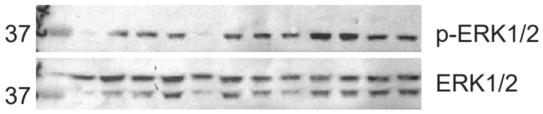

p-ERK1/2 and ERK1/2 were detected on the same membrane

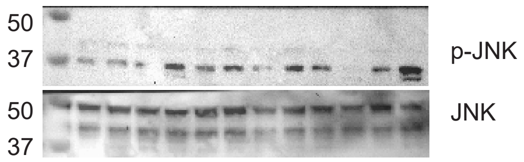

p-JNK and JNK were detected on the same membrane

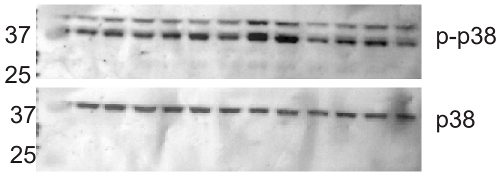

p-p38 and p38 were detected on the same membrane
