# Supplementary material for: Cyclic fasting bolsters cholesterol biosynthesis inhibitors’ anticancer activity
Source: Nat Commun. 2023 Oct 31;14:6951. doi: 10.1038/s41467-023-42652-1 (PMC10618279; doi:10.1038/s41467-023-42652-1)
Supplement: Supplementary file 2 — Reporting Summary [file 41467_2023_42652_MOESM2_ESM.pdf]

nature portfolio

Corresponding author(s): Dr. Alessio Nencioni, Dr.ssa Irene Caffa

Last updated by author(s): Sep 21, 2023

Reporting Summary

Nature Portfolio wishes to improve the reproducibility of the work that we publish. This form provides structure for consistency and transparency in reporting. For further information on Nature Portfolio policies, see our Editorial Policies and the Editorial Policy Checklist.

Statistics

For all statistical analyses, confirm that the following items are present in the figure legend, table legend, main text, or Methods section.

n/a	Confirmed	
☐	☒	The exact sample size (*n*) for each experimental group/condition, given as a discrete number and unit of measurement
☐	☒	A statement on whether measurements were taken from distinct samples or whether the same sample was measured repeatedly
☐	☒	The statistical test(s) used AND whether they are one- or two-sided<br>*Only common tests should be described solely by name; describe more complex techniques in the Methods section.*
☐	☒	A description of all covariates tested
☐	☒	A description of any assumptions or corrections, such as tests of normality and adjustment for multiple comparisons
☐	☒	A full description of the statistical parameters including central tendency (e.g. means) or other basic estimates (e.g. regression coefficient) AND variation (e.g. standard deviation) or associated estimates of uncertainty (e.g. confidence intervals)
☐	☒	For null hypothesis testing, the test statistic (e.g. *F*, *t*, *r*) with confidence intervals, effect sizes, degrees of freedom and *P* value noted<br>*Give P values as exact values whenever suitable.*
☒	☐	For Bayesian analysis, information on the choice of priors and Markov chain Monte Carlo settings
☒	☐	For hierarchical and complex designs, identification of the appropriate level for tests and full reporting of outcomes
☒	☐	Estimates of effect sizes (e.g. Cohen's *d*, Pearson's *r*), indicating how they were calculated

Our web collection on statistics for biologists contains articles on many of the points above.

Software and code

Policy information about availability of computer code

Data collection	Band intensities of Western Blots were detected by Quantity One 4.6.6 SW software (Bio-Rad Laboratories, Inc.).<br>Mouse blood counts and measurement of serum biochemical parameters were detected with the Ilab-650 automatic instrument software 1.01 (Werfen).<br>Oxygen consumption was detected with Sensor Trace software (Unisense Microrespiration).<br>Cholesterol efflux was detected with QuantaSmart TM 5.2 software (Perkin Elmer).
Data analysis	Statistical analyses were performed with GraphPad Prism software version 8.0 (San Diego, CA, USA).

For manuscripts utilizing custom algorithms or software that are central to the research but not yet described in published literature, software must be made available to editors and reviewers. We strongly encourage code deposition in a community repository (e.g. GitHub). See the Nature Portfolio guidelines for submitting code & software for further information.

Data

Policy information about availability of data

All manuscripts must include a data availability statement. This statement should provide the following information, where applicable:

- Accession codes, unique identifiers, or web links for publicly available datasets
- A description of any restrictions on data availability
- For clinical datasets or third party data, please ensure that the statement adheres to our policy

Source data for all figures and supplementary information are provided with the paper.

Human research participants

Policy information about studies involving human research participants and Sex and Gender in Research.

Reporting on sex and gender	N/A
Population characteristics	N/A
Recruitment	N/A
Ethics oversight	N/A

Note that full information on the approval of the study protocol must also be provided in the manuscript.

Field-specific reporting

Please select the one below that is the best fit for your research. If you are not sure, read the appropriate sections before making your selection.

☒ Life sciences ☐ Behavioural & social sciences ☐ Ecological, evolutionary & environmental sciences

For a reference copy of the document with all sections, see nature.com/documents/nr-reporting-summary-flat.pdf

Life sciences study design

All studies must disclose on these points even when the disclosure is negative.

Sample size	For our in vitro experiments, no statistical method was used to predetermine sample size, but our sample size were similar to those reported in several publication including ours (at least three technical replicates each experiment).<br>For our in vivo experiments, we estimated sample size by PS (Power and Sample size calculation-Vanderbilt University) software considering a multifactorial variance analysis. By this approach we estimated that the minimum number of mice that was assigned to each treatment group in our in vivo experiments would reach a power of 0.85. The Type I error probability associated with our tests of the null hypothesis was 0.05.
Data exclusions	No data were excluded
Replication	We confirm that all in vitro experiments were reproducible by repeating at least three times and by using different lots of reagents, different stocks of cell lines and some experiments were also repeated and confirmed by other operators.
Randomization	Samples and mice were assigned to the different experimental groups in a random fashion.
Blinding	Operators were unblinded during in vitro experiments (cells viability, colony formation assay, WB, qPCR, cholesterol quantification and cholesterol efflux measurement) since cells were treated with different media to mimic fasting condition.<br>Operators were blinded during oxidative phosphorylation measurement since we provided them tumour masses labeled with a group code.<br>Blinding during animal experiments was not possible because mice underwent a specific diet (i.e. ,water-only fasting) and daily treatment.

Behavioural & social sciences study design

All studies must disclose on these points even when the disclosure is negative.

Study description	*Briefly describe the study type including whether data are quantitative, qualitative, or mixed-methods (e.g. qualitative cross-sectional, quantitative experimental, mixed-methods case study).*

Research sample	*State the research sample (e.g. Harvard university undergraduates, villagers in rural India) and provide relevant demographic information (e.g. age, sex) and indicate whether the sample is representative. Provide a rationale for the study sample chosen. For studies involving existing datasets, please describe the dataset and source.*
Sampling strategy	*Describe the sampling procedure (e.g. random, snowball, stratified, convenience). Describe the statistical methods that were used to predetermine sample size OR if no sample-size calculation was performed, describe how sample sizes were chosen and provide a rationale for why these sample sizes are sufficient. For qualitative data, please indicate whether data saturation was considered, and what criteria were used to decide that no further sampling was needed.*
Data collection	*Provide details about the data collection procedure, including the instruments or devices used to record the data (e.g. pen and paper, computer, eye tracker, video or audio equipment) whether anyone was present besides the participant(s) and the researcher, and whether the researcher was blind to experimental condition and/or the study hypothesis during data collection.*
Timing	*Indicate the start and stop dates of data collection. If there is a gap between collection periods, state the dates for each sample cohort.*
Data exclusions	*If no data were excluded from the analyses, state so OR if data were excluded, provide the exact number of exclusions and the rationale behind them, indicating whether exclusion criteria were pre-established.*
Non-participation	*State how many participants dropped out/declined participation and the reason(s) given OR provide response rate OR state that no participants dropped out/declined participation.*
Randomization	*If participants were not allocated into experimental groups, state so OR describe how participants were allocated to groups, and if allocation was not random, describe how covariates were controlled.*

Ecological, evolutionary & environmental sciences study design

All studies must disclose on these points even when the disclosure is negative.

Study description	*Briefly describe the study. For quantitative data include treatment factors and interactions, design structure (e.g. factorial, nested, hierarchical), nature and number of experimental units and replicates.*
Research sample	*Describe the research sample (e.g. a group of tagged Passer domesticus, all Stenocereus thurberi within Organ Pipe Cactus National Monument), and provide a rationale for the sample choice. When relevant, describe the organism taxa, source, sex, age range and any manipulations. State what population the sample is meant to represent when applicable. For studies involving existing datasets, describe the data and its source.*
Sampling strategy	*Note the sampling procedure. Describe the statistical methods that were used to predetermine sample size OR if no sample-size calculation was performed, describe how sample sizes were chosen and provide a rationale for why these sample sizes are sufficient.*
Data collection	*Describe the data collection procedure, including who recorded the data and how.*
Timing and spatial scale	*Indicate the start and stop dates of data collection, noting the frequency and periodicity of sampling and providing a rationale for these choices. If there is a gap between collection periods, state the dates for each sample cohort. Specify the spatial scale from which the data are taken*
Data exclusions	*If no data were excluded from the analyses, state so OR if data were excluded, describe the exclusions and the rationale behind them, indicating whether exclusion criteria were pre-established.*
Reproducibility	*Describe the measures taken to verify the reproducibility of experimental findings. For each experiment, note whether any attempts to repeat the experiment failed OR state that all attempts to repeat the experiment were successful.*
Randomization	*Describe how samples/organisms/participants were allocated into groups. If allocation was not random, describe how covariates were controlled. If this is not relevant to your study, explain why.*
Blinding	*Describe the extent of blinding used during data acquisition and analysis. If blinding was not possible, describe why OR explain why blinding was not relevant to your study.*

Did the study involve field work? ☐ Yes ☐ No

Field work, collection and transport

Field conditions	*Describe the study conditions for field work, providing relevant parameters (e.g. temperature, rainfall).*
Location	*State the location of the sampling or experiment, providing relevant parameters (e.g. latitude and longitude, elevation, water depth).*

Access & import/export	*Describe the efforts you have made to access habitats and to collect and import/export your samples in a responsible manner and in compliance with local, national and international laws, noting any permits that were obtained (give the name of the issuing authority, the date of issue, and any identifying information).*
Disturbance	*Describe any disturbance caused by the study and how it was minimized.*

Reporting for specific materials, systems and methods

We require information from authors about some types of materials, experimental systems and methods used in many studies. Here, indicate whether each material, system or method listed is relevant to your study. If you are not sure if a list item applies to your research, read the appropriate section before selecting a response.

Materials & experimental systems

n/a	Involved in the study
☐	☒ Antibodies
☐	☒ Eukaryotic cell lines
☒	☐ Palaeontology and archaeology
☐	☒ Animals and other organisms
☒	☐ Clinical data
☒	☐ Dual use research of concern

Methods

n/a	Involved in the study
☒	☐ ChIP-seq
☐	☒ Flow cytometry
☒	☐ MRI-based neuroimaging

Antibodies

Antibodies used

Anti-phospho-AKT(Ser473;clone193H12, Cat Num #4058s, Lot 30, diluted 1:1000, Cell Signaling Technology, Danvers, MA, USA),
anti-AKT (Cat Num #9272s, Lot 25, diluted 1:1000, Cell Signaling Technology, Danvers, MA, USA),
anti-phospho SAPK/JNK (Thr183/Tyr185; clone G9, Cat Num#9255, Lot 35 diluted 1:2000, Cell Signaling Technology, Danvers, MA, USA),
anti-SAPK/JNK (Cat Num #9252, Lot 18, diluted 1:1000, Cell Signaling Technology, Danvers, MA, USA),
anti-phospho-p44/42 MAPK (Erk1/2) (Thr202/Tyr204; clone197G2, Cat Num#4377 Lot 10, diluted 1:100, Cell Signaling Technology, Danvers, MA, USA),
anti-Erk1/2 (Cat Num#9102, Lot 23, diluted 1:1000, Cell Signaling Technology, Danvers, MA, USA),
anti-phospho-p38 MAPK (Thr180/Tyr182;clone 12F8, Cat Num#4631, Lot 9, dilution 1:1000 Cell Signaling Technology, Danvers, MA, USA),
anti-p38 MAPK (cloneD13E1, Cat Num# 8690, Lot 9, diluted 1:1000, Cell Signaling Technology, Danvers, MA, USA),
anti-caveolin-1 (clone D46G3, Cat Num#3267, diluted 1:1000, Cell Signaling Technology, Danvers, MA, USA),
anti-SQLE (Cat Num#40659, Lot 1, diluted 1:1000, Cell Signaling Technology, Danvers, MA, USA),
anti-DHCR24 (cloneC59D8; Cat Num#2033, Lot 2, diluted 1:1000, Cell Signaling Technology, Danvers, MA, USA),
anti-ABCA1 (cloneE7X5G; Cat Num#96292, Lot 1, diluted 1:1000, Cell Signaling Technology, Danvers, MA, USA),
anti-phospho STAT3 (clone B7; Tyr705; Cat Num sc-8059, Lot J0813, diluted 1:200, Santa Cruz Biotechnology),
anti-STAT3 (clone C20; Cat Num sc-482, Lot C2414, diluted 1:200, Santa Cruz Biotechnology),
anti-β-actin (Cat Num sc47778, Lot F0215, diluted 1:10000, Santa Cruz Biotechnology).
anti-Cyclophilin B (Cat Num #PA1-027A, Lot WH338304, diluted 1:1000, Thermo Fisher).
anti-ABCG1 (Cat Num#13578-1-AP, Lot 00057963, diluted 1:1000, Proteintech).
anti-LDLR (Cat Num#10785-1-AP, Lot 00118476, diluted 1:1000, Proteintech).
anti-Rabbit (Cat Num sc-2357, Lot Num A0422, diluted 1:5000, Santa Cruz Biotechnology).
anti-Mouse (Cat Num sc-516102, Lot Num F1422, diluted 1:5000, Santa Cruz Biotechnology).

Validation

phospho-AKT antibody was validated on NIH/3T3 cells treated with PDGF. Relevant citations:A MST1-FOXO1 cascade establishes endothelial tip cell polarity and facilitates sprouting angiogenesis. Nature Communications, 2019 by Kim, Y. H. et al..
The N6-methyladenosine (m6A)-forming enzyme METTL3 controls myeloid differentiation of normal hematopoietic and leukemia cells. Nature Medicine, 2017 by Vu, L. P. et al..

anti-AKT was validated on MCF7 cells transduced with Akt Myr. Relevant citations:Identification of the PTEN-ARID4B-PI3K pathway reveals the dependency on ARID4B by PTEN-deficient prostate cancer. Nature Communications, 2019 by Wu, R. C. et al.
Patient-derived lung cancer organoids as in vitro cancer models for therapeutic screening. Nature Communications, 2019 by Kim, M. et al.

anti-phospho SAPK/JNK was validated on NIH/3T3 cells treated with UV. Relevant citations:Dynamic changes of muscle insulin sensitivity after metabolic surgery. Nature Communications, 2019 by Gancheva, S et. al.
Transcriptional regulation of autophagy-lysosomal function in BRAF-driven melanoma progression and chemoresistance. Nature Communications, 2019 by Li, S et. al.

anti-SAPK/JNK was validated on 293 cells. Relevant citations: TRIM25 promotes the cell survival and growth of hepatocellular carcinoma through targeting Keap1-Nrf2 pathway Nature Communications, 2020 by Liu, Y et. al.
Dynamic changes of muscle insulin sensitivity after metabolic surgery. Nature Communications, 2019 by Gancheva, S et. al.

anti-phospho-p44/42 MAPK was validated on NIH/3T3 cells treated with UV+PDGF. Relevant citations: A proximity biotinylation-based approach to identify protein-E3 ligase interactions induced by PROTACs and molecular glues. Nature Communications, 2022 by Yamanaka, S et. al.
A human immunodeficiency syndrome caused by mutations in CARMIL2. Nature Communications, 2017 by Schober, T et. al.

anti-Erk1/2 was validated on Hela cells. Relevant citations: Targeting the latent human cytomegalovirus reservoir for T-cell-mediated killing with virus-specific nanobodies. Nature Communications, 2021 by De Groof, T et. al.
Selective and noncovalent targeting of RAS mutants for inhibition and degradation. Nature Communications, 2021 by Teng, KW et. al.

anti-phospho-p38 MAPK was validated on NIH/3T3 cells. Relevant citations: In vitro anti-inflammatory effects of AZD8999, a novel bifunctional muscarinic acetylcholine receptor antagonist /β2-adrenoceptor agonist (MABA) compound in neutrophils from COPD patients. PlosOne, 2019 Milara, J et. al.
IGFBP2 enhances adipogenic differentiation potentials of mesenchymal stem cells from Wharton's jelly of the umbilical cord via JNK and Akt signaling pathways. PlosOne, 2017 Wang, J et. al.

anti-p38 MAPK was validated on NIH/3T3 cells. Relevant citations: EZH2 engages TGFβ signaling to promote breast cancer bone metastasis via integrin β1-FAK activation. Nature Communications, 2022 by Zhang, L et. al.
Enhancement of anaerobic glycolysis - a role of PGC-1α4 in resistance exercise. Nature Communications, 2022 by Koh, J et. al.

anti-caveolin-1 was validated on Hela cells. Relevant citations: MT4-MMP deficiency increases patrolling monocyte recruitment to early lesions and accelerates atherosclerosis. Nature Communications, 2018 byClemente, C et. al.
Cigarette smoke increases susceptibility to infection in lung epithelial cells by upregulating caveolin-dependent endocytosis. PlosOne, 2020 Duffney, PF et. al.

anti-SQLE was validated on Hep G2 cells. Relevant citations: Mitochondrial respiratory chain dysfunction alters ER sterol sensing and mevalonate pathway activity. J Biol Chem, 2022 Wall, J et. al.

anti-DHCR24 was validated on MCF7 cells. Relevant citations: MMAB promotes negative feedback control of cholesterol homeostasis. Nature Communications, 2021 by Goedeke, L et. al.
NPC1 Confers Metabolic Flexibility in Triple Negative Breast Cancer. Cancers (Basels), 2022 by O'Neill, K et. al.

anti-ABCA1 was validated on Hep G2 cells. Relevant citations: Mutant APC reshapes Wnt signaling plasma membrane nanodomains by altering cholesterol levels via oncogenic β-catenin. Nature Communications, 2023 by Erazo-Oliveras, A et. al.
Ferredoxin reductase and p53 are necessary for lipid homeostasis and tumor suppression through the ABCA1-SREBP pathway. Oncogene, 2022 by Zhang, Y et. al.

anti-phospho STAT3 was validated on 3T3-L1 cells. Relevant citations: Disrupted metabolic signatures in amniotic fluid associated with increased risk of intestinal inflammation in cesarean section offspring. Front. Immunol. 2022 Liu, Y., et al.

anti-STAT3 was vaidated on cells transduced with STAT3. Relevant citations: Systemic Lipopolysaccharide Challenge Induces Inflammatory Changes in Rat Dorsal Root Ganglia: An Ex Vivo Study. Int J Mol Sci. 2022 Nürnberger, F., et al

anti-β-actin was validated on NIH/3T3 cells. Relevant citations: B1 oligomerization regulates PML nuclear body biogenesis and leukemogenesis. Nature Communications 2019, Li Y, et al.
Transcriptional regulation of autophagy-lysosomal function in BRAF-driven melanoma progression and chemoresistance. Nature Communications, 2019 Li S, et al.

anti-Cyclophilin B was validated on MCF7 cells. Relevant citations: Mitofusin 1 and 2 regulation of mitochondrial DNA content is a critical determinant of glucose homeostasis. Nature Communications, 2022 Sidarala V, et al.
TAPBPR promotes antigen loading on MHC-I molecules using a peptide trap. Nature Communications, 2021 McShan AC, et al.

anti-ABCG1 was validated on Hela cells. Relevant citations: Metabolic effects of RUBCN/Rubicon deficiency in kidney proximal tubular epithelial cells. Autophagy, 2020 Matsuda J, et al.

anti-LDLR was validated on Hela cells. Relevant citations: A genome-wide CRISPR screen identifies host factors that regulate SARS-CoV-2 entry. Nature Communications, 2021 Zhu Y, et al.
Elevation of JAML Promotes Diabetic Kidney Disease by Modulating Podocyte Lipid Metabolism. Cell Metab. 2020 Fu Y.

Eukaryotic cell lines

Policy information about cell lines and Sex and Gender in Research

Cell line source(s)

Capan-1 (catalog number HTB-79), MIA PaCa-2 (catalog number CRL-1420), PANC-1 (catalog number CRL-1469), BxPC-3 (catalog number CRL-1687), HCT116 (catalog number CCL-247), HT29 (catalog number HTB-38, CT26 (catalog number CRL-2638), N87 (catalog number CRL-5822), PC3 (catalog number CRL-1435), MCF7 (catalog number HTB-22), MDA-MB-231 (catalog number HTB-26), SKBR3 (catalog number HTB-30) , 4T1 (catalog number CRL-2539), H1975 (catalog number CRL-5908), B16 (catalog number CRL-6322), HPNE (catalog number CRL-4023) and A549 (catalog number CCL-185) cell lines were purchased from the ATCC (LGC Standards S.r.l., Milan, Italy). ID8 cells (mouse ovarian cancer; catalog number SCC145) were purchased from Sigma Aldrich S.r.l. (Italy). PK9 PDAC cells (Ref. 59) were kindly provided by Dr. Georg Feldmann (University Hospital of Bonn, Bonn, Germany). OVCAR5 and OVCAR8 ovarian cancer cells (from the Developmental

	Therapeutics Program of the National Cancer Institute, Bethesda, MD, USA) were a kind gift of Dr. Gabriele Zoppoli (Department of Internal Medicine and Medical Specialties, University of Genoa).
Authentication	Cells were authenticated by DNA fingerprinting and isozyme detection.
Mycoplasma contamination	All cell lines were routinely tested for Mycoplasma contamination and results were always negative.
Commonly misidentified lines (See ICLAC register)	None of the cell lines used in our study belongs to the commonly misidentified lines.

Palaeontology and Archaeology

Specimen provenance	*Provide provenance information for specimens and describe permits that were obtained for the work (including the name of the issuing authority, the date of issue, and any identifying information). Permits should encompass collection and, where applicable, export.*
Specimen deposition	*Indicate where the specimens have been deposited to permit free access by other researchers.*
Dating methods	*If new dates are provided, describe how they were obtained (e.g. collection, storage, sample pretreatment and measurement), where they were obtained (i.e. lab name), the calibration program and the protocol for quality assurance OR state that no new dates are provided.*

☐ Tick this box to confirm that the raw and calibrated dates are available in the paper or in Supplementary Information.

Ethics oversight	*Identify the organization(s) that approved or provided guidance on the study protocol, OR state that no ethical approval or guidance was required and explain why not.*

Note that full information on the approval of the study protocol must also be provided in the manuscript.

Animals and other research organisms

Policy information about studies involving animals; ARRIVE guidelines recommended for reporting animal research, and Sex and Gender in Research

Laboratory animals	6-8 weeks old female Athymic nude mice and 6-8 weeks old female C57BL/6 mice were utilized in our animal experiments.
Wild animals	The study did not involve wild animals
Reporting on sex	Only one sex was used in the individual mouse experiments. The overall numbers were provided in the results.
Field-collected samples	The study did not involve field-collected samples.
Ethics oversight	Animal work was only started upon approval by the Italian Istituto Superiore di Sanità (ISS) with the authorization n°280/2022, protocol 22418.169

Note that full information on the approval of the study protocol must also be provided in the manuscript.

Clinical data

Policy information about clinical studies

All manuscripts should comply with the ICMJEguidelines for publication of clinical research and a completedCONSORT checklist must be included with all submissions.

Clinical trial registration	*Provide the trial registration number from ClinicalTrials.gov or an equivalent agency.*
Study protocol	*Note where the full trial protocol can be accessed OR if not available, explain why.*
Data collection	*Describe the settings and locales of data collection, noting the time periods of recruitment and data collection.*
Outcomes	*Describe how you pre-defined primary and secondary outcome measures and how you assessed these measures.*

Dual use research of concern

Policy information about dual use research of concern

Hazards

Could the accidental, deliberate or reckless misuse of agents or technologies generated in the work, or the application of information presented in the manuscript, pose a threat to:

No	Yes	
☐	☐	Public health
☐	☐	National security
☐	☐	Crops and/or livestock
☐	☐	Ecosystems
☐	☐	Any other significant area

Experiments of concern

Does the work involve any of these experiments of concern:

No	Yes	
☐	☐	Demonstrate how to render a vaccine ineffective
☐	☐	Confer resistance to therapeutically useful antibiotics or antiviral agents
☐	☐	Enhance the virulence of a pathogen or render a nonpathogen virulent
☐	☐	Increase transmissibility of a pathogen
☐	☐	Alter the host range of a pathogen
☐	☐	Enable evasion of diagnostic/detection modalities
☐	☐	Enable the weaponization of a biological agent or toxin
☐	☐	Any other potentially harmful combination of experiments and agents

ChIP-seq

Data deposition

☐ Confirm that both raw and final processed data have been deposited in a public database such as GEO.

☐ Confirm that you have deposited or provided access to graph files (e.g. BED files) for the called peaks.

Data access links<br>*May remain private before publication.*	*For "Initial submission" or "Revised version" documents, provide reviewer access links. For your "Final submission" document, provide a link to the deposited data.*
Files in database submission	*Provide a list of all files available in the database submission.*
Genome browser session<br>(e.g. UCSC)	*Provide a link to an anonymized genome browser session for "Initial submission" and "Revised version" documents only, to enable peer review. Write "no longer applicable" for "Final submission" documents.*

Methodology

Replicates	*Describe the experimental replicates, specifying number, type and replicate agreement.*
Sequencing depth	*Describe the sequencing depth for each experiment, providing the total number of reads, uniquely mapped reads, length of reads and whether they were paired- or single-end.*
Antibodies	*Describe the antibodies used for the ChIP-seq experiments; as applicable, provide supplier name, catalog number, clone name, and lot number.*
Peak calling parameters	*Specify the command line program and parameters used for read mapping and peak calling, including the ChIP, control and index files used.*
Data quality	*Describe the methods used to ensure data quality in full detail, including how many peaks are at FDR 5% and above 5-fold enrichment.*
Software	*Describe the software used to collect and analyze the ChIP-seq data. For custom code that has been deposited into a community repository, provide accession details.*

Flow Cytometry

Plots

Confirm that:

☒ The axis labels state the marker and fluorochrome used (e.g. CD4-FITC).

☒ The axis scales are clearly visible. Include numbers along axes only for bottom left plot of group (a 'group' is an analysis of identical markers).

☒ All plots are contour plots with outliers or pseudocolor plots.

☒ A numerical value for number of cells or percentage (with statistics) is provided.

Methodology

Sample preparation	Peripheral blood mononuclear cells were stained with 5 ul Annexin-V-FITC (Thermofisher Scientific) and 2 ug/ml propidium iodide in 100 ul AV binding buffer (Thermofisher) according to the instructions of the Annexin V's manufacturer. Thereafter 400 ul AV binding buffer was added and cells were analysed by flow cytometry by acquiring 10,000 events per treatment condition.
Instrument	FACS Calibur (Becton Dickinson, Milan, Italy).
Software	Data were processed by Cellquest analysis software (Beckman coulter, version 2.0).
Cell population abundance	All populations were analyzed without post-sort analysis.
Gating strategy	The analysis of Annexin-V and propidium iodide fluorescence in PBMCs was done by gating on the lymphocyte population. A figure exemplifying the gating strategy is presented in the Supplementary Information.

☐ Tick this box to confirm that a figure exemplifying the gating strategy is provided in the Supplementary Information.

Magnetic resonance imaging

Experimental design

Design type	*Indicate task or resting state; event-related or block design.*
Design specifications	*Specify the number of blocks, trials or experimental units per session and/or subject, and specify the length of each trial or block (if trials are blocked) and interval between trials.*
Behavioral performance measures	*State number and/or type of variables recorded (e.g. correct button press, response time) and what statistics were used to establish that the subjects were performing the task as expected (e.g. mean, range, and/or standard deviation across subjects).*

Acquisition

Imaging type(s)	*Specify: functional, structural, diffusion, perfusion.*
Field strength	*Specify in Tesla*
Sequence & imaging parameters	*Specify the pulse sequence type (gradient echo, spin echo, etc.), imaging type (EPI, spiral, etc.), field of view, matrix size, slice thickness, orientation and TE/TR/flip angle.*
Area of acquisition	*State whether a whole brain scan was used OR define the area of acquisition, describing how the region was determined.*

Diffusion MRI ☐ Used ☐ Not used

Preprocessing

Preprocessing software	*Provide detail on software version and revision number and on specific parameters (model/functions, brain extraction, segmentation, smoothing kernel size, etc.).*
Normalization	*If data were normalized/standardized, describe the approach(es): specify linear or non-linear and define image types used for transformation OR indicate that data were not normalized and explain rationale for lack of normalization.*
Normalization template	*Describe the template used for normalization/transformation, specifying subject space or group standardized space (e.g. original Talairach, MNI305, ICBM152) OR indicate that the data were not normalized.*
Noise and artifact removal	*Describe your procedure(s) for artifact and structured noise removal, specifying motion parameters, tissue signals and physiological signals (heart rate, respiration).*

Volume censoring | *Define your software and/or method and criteria for volume censoring, and state the extent of such censoring.*

Statistical modeling & inference

Model type and settings | *Specify type (mass univariate, multivariate, RSA, predictive, etc.) and describe essential details of the model at the first and second levels (e.g. fixed, random or mixed effects; drift or auto-correlation).*

Effect(s) tested | *Define precise effect in terms of the task or stimulus conditions instead of psychological concepts and indicate whether ANOVA or factorial designs were used.*

Specify type of analysis: ☐ Whole brain ☐ ROI-based ☐ Both

Statistic type for inference (See Eklund et al. 2016) | *Specify voxel-wise or cluster-wise and report all relevant parameters for cluster-wise methods.*

Correction | *Describe the type of correction and how it is obtained for multiple comparisons (e.g. FWE, FDR, permutation or Monte Carlo).*

Models & analysis

n/a	Involved in the study
☐	☐ Functional and/or effective connectivity
☐	☐ Graph analysis
☐	☐ Multivariate modeling or predictive analysis

Functional and/or effective connectivity | *Report the measures of dependence used and the model details (e.g. Pearson correlation, partial correlation, mutual information).*

Graph analysis | *Report the dependent variable and connectivity measure, specifying weighted graph or binarized graph, subject- or group-level, and the global and/or node summaries used (e.g. clustering coefficient, efficiency, etc.).*

Multivariate modeling and predictive analysis | *Specify independent variables, features extraction and dimension reduction, model, training and evaluation metrics.*
