# Supplementary material for: Cyclic fasting bolsters cholesterol biosynthesis inhibitors’ anticancer activity
Source: Nat Commun. 2023 Oct 31;14:6951. doi: 10.1038/s41467-023-42652-1 (PMC10618279; doi:10.1038/s41467-023-42652-1)
Supplement: Supplementary file 3 — Description of Additional Supplementary Files [file 41467_2023_42652_MOESM3_ESM.pdf]

Title: Supplementary Movie 1

Description: Patient-derived colorectal cancer organoids (OMCR15-045TK) were treated w/ or w/o clotrimazole (15 µM), terbinafine (20 µM), FMCC or their combination and imaged three times a day to generate a time-lapse for every treatment condition. One representative well per type of treatment is shown.
